# Efficacy of Web-Based Collection of Strength-Based Testimonials for Text Message Extension of Youth Suicide Prevention Program: Randomized Controlled Experiment

**DOI:** 10.2196/publichealth.6207

**Published:** 2016-11-09

**Authors:** Phyo Thiha, Anthony R Pisani, Kunali Gurditta, Erin Cherry, Derick R Peterson, Henry Kautz, Peter A Wyman

**Affiliations:** ^1^ Department of Computer Science University of Rochester Rochester, NY United States; ^2^ Department of Psychiatry University of Rochester School of Medicine and Dentistry Rochester, NY United States; ^3^ Department of Pediatrics University of Rochester School of Medicine and Dentistry Rochester, NY United States; ^4^ Northrop Grumman Huntsville, AL United States; ^5^ Department of Biostatistics and Computational Biology University of Rochester Rochester, NY United States

**Keywords:** mental health, adolescent health, user interface design, suicide, text messaging

## Abstract

**Background:**

Equipping members of a target population to deliver effective public health messaging to peers is an established approach in health promotion. The Sources of Strength program has demonstrated the promise of this approach for “upstream” youth suicide prevention. Text messaging is a well-established medium for promoting behavior change and is the dominant communication medium for youth. In order for peer ‘opinion leader’ programs like Sources of Strength to use scalable, wide-reaching media such as text messaging to spread peer-to-peer messages, they need techniques for assisting peer opinion leaders in creating effective testimonials to engage peers and match program goals. We developed a Web interface, called Stories of Personal Resilience in Managing Emotions (StoryPRIME), which helps peer opinion leaders write effective, short-form messages that can be delivered to the target population in youth suicide prevention program like Sources of Strength.

**Objective:**

To determine the efficacy of StoryPRIME, a Web-based interface for remotely eliciting high school peer leaders, and helping them produce high-quality, personal testimonials for use in a text messaging extension of an evidence-based, peer-led suicide prevention program.

**Methods:**

In a double-blind randomized controlled experiment, 36 high school students wrote testimonials with or without eliciting from the StoryPRIME interface. The interface was created in the context of Sources of Strength–an evidence-based youth suicide prevention program–and 24 ninth graders rated these testimonials on relatability, usefulness/relevance, intrigue, and likability.

**Results:**

Testimonials written with the StoryPRIME interface were rated as more relatable, useful/relevant, intriguing, and likable than testimonials written without StoryPRIME, *P*=.054.

**Conclusions:**

StoryPRIME is a promising way to elicit high-quality, personal testimonials from youth for prevention programs that draw on members of a target population to spread public health messages.

## Introduction

### Background and Motivation

Public health practitioners and researchers are increasingly harnessing mobile technology to enhance the reach and effectiveness of evidence-based interventions [1-3]. Equipping members of a target population to deliver effective public health messaging to peers is an established approach in substance abuse prevention, human immunodeficiency virus prevention, and health promotion [4-7], and is promising for suicide prevention [8]. The effectiveness of peer-to-peer public health messaging strategies is congruent with peer-to-peer influences on behavior [10]. For youth with acute distress and with numerous risk factors for suicide, social modeling of emotional regulation strategies could slow or reverse a negative trajectory or prevent impulsive attempts to escape painful emotions [11,12]. A crucial barrier to technological extensions of these so-called ‘opinion leader’ interventions is the challenge of helping peer leaders to deliver effective messages to peers remotely. This requires development of Web-based replacements for typical in-person components [9]. In this study, we tested a Web-based interface for remotely helping high school peer leaders to produce high-quality, personal testimonials for use in a text messaging extension of an evidence-based, peer-led suicide prevention program.

Sources of Strength is a school-based suicide prevention program that is certified on the United States National Registry of Evidence-Based Programs and Practices. The program seeks to reduce risk and strengthen protective factors in the population by preparing diverse ‘key opinion leaders’ to conduct public health messaging and activities with peers to increase school-wide positive coping norms and communication with trusted adults, and bring suicidal peers to seek for adult help [8,13]. Currently implemented in over 3000 high schools and colleges across the United States, Sources of Strength was the first suicide prevention program to apply a peer ‘opinion leader’ approach. As part of this strategy, peer leaders publicly share personal stories (or testimonials) of “hope, help, and strength” with peers and encourage them to do the same. Those who share their personal stories act as distributed change agents, generating much of the intervention content received by their school’s population. In a randomized controlled trial conducted in 18 schools, trained peer leaders were 4 times more likely to refer a suicidal friend to an adult, and school-wide help-seeking norms improved in schools implementing Sources of Strength [8]. An ongoing trial with 40 schools in rural and underserved communities is testing the impact and proposed network diffusion model of Sources of Strength on reducing suicide attempts.

Text4Strength is a text messaging extension of Sources of Strength. It is currently under development with funding from the National Institute of Mental Health (K23MH101449). Text4Strength uses text-based peer testimonials and other types of text messages to introduce high school students to Sources of Strength concepts and extends Sources of Strength by also introducing emotional skills associated with decreased depressive symptoms and lower suicide risk [14-17]. Text messaging is a logical medium for disseminating messages because it is the dominant mode of communication for youth [18] and has strong empirical support for delivering health interventions [2,3,19-25]. The current version of Text4Strength specifically targets ninth graders because research indicates an increase in emotional and behavioral problems between ages 14 and 15, when most students are starting high school [26]. In addition to normal developmental changes resulting in heightened emotional activation [27-29] and diminished inhibitory control abilities [30], ninth graders face new challenges associated with the transition to high school, including greater responsibility for seeking help, greater emphasis on romantic relationships, and greater academic pressure.

In developing Text4Strength, we faced challenges that previous work in translating behavioral interventions into text (e.g., [9]) had not yet surmounted: collecting interesting, personal, and credible [31,32] testimonials consistent with the intervention model, goals, and targets. We addressed these challenges by developing Stories of Personal Resilience in Managing Emotions (StoryPRIME), a Web-based interface to guide peer leaders through the process of remembering, sharing, and condensing relevant personal stories into the pithy testimonials needed for text messaging. We developed StoryPRIME through an interdisciplinary design process in which suicide prevention researchers consulted with human-computer interaction (HCI) researchers. Together, we conducted design workgroups with high school peer leaders, as well as feasibility testing with an adult sample to ensure the safety of the StoryPRIME interface prior to testing it in a randomized controlled experiment with a teenage population. In this paper, we report on results from the randomized controlled experiment of StoryPRIME to discover whether or not Web-based eliciting could assist peer leaders in generating high-quality text message testimonials that are interesting, personal, and credible to ninth graders.

### Development and Feasibility Testing

Suicide prevention and HCI researchers worked together to translate practices used in Sources of Strength to elicit peer leaders to tell their stories using a Web-based interface. In the Sources of Strength training program, student peer leaders participate in a 4-hour training in which helpful testimonials are modeled and practiced. After initial training, students participate in approximately biweekly meetings where adult advisors give them opportunities to practice and receive feedback sharing their “hope, help, and strength” stories with peers and encourage them to do the same [13]. The challenge we faced was translating this highly organic discussion and coaching process in Sources of Strength into a structured, Web-based interface.

To tackle this challenge, HCI researchers piloted an initial interface with our interdis­ciplinary team and other lab members, and then conducted a participatory design [33] session and workgroup with 7 high school peer leaders (4 females, 3 males). We met with the students 7 times, for 60-90 minutes each, to get their feedback on the design of the StoryPRIME interface for testimonial writing and in general, about our plans for the Text4Strength text messaging program. Using this feedback, HCI researchers developed several variations/prototypes of the StoryPRIME interface. We then asked the workgroup participants to write stories about themselves using these interfaces, and discuss the writing experience with the workgroup participants and our research group. Based on the workgroup’s feedback, we refined the StoryPRIME interface, partitioning the writing process into 3 main steps to help writers focus on the essential parts of the story: (1) challenge faced, (2) how they solved it, and (3) the outcome.

We pilot tested the StoryPRIME interface with an adult convenience sample in order to ensure feasibility and safety before testing with adolescent high school students. Fifty adults (29 females, 21 males; mean age of 30.28 (standard deviation=7.73)) were recruited from Mechanical Turk (MTurk)–a crowdsourcing website where people are paid to do Web-based tasks. Participants wrote testimonials about com­mon stressful situations and emotional skills and resources they used to cope. A separate sample of 61 adults recruited from MTurk rated the testimonials on how interesting, personal, genuine/credible, and relatable they were. In addition, 2 public health experts rated the stories on how appropriate and (potentially) effective they were. The results were promising: 70% (35/50) of the testimonials were judged as interesting, 78% (39/50) as personal, 80% (40/50) as credible, and 76% (38/50) as relatable. The two suicide prevention experts judged 76% (38/50) and 72% (36/50) of the testimonials as appropriate/safe and effective, respectively. Furthermore, qualitative responses from Web-based participants indicated that they found the experience rewarding and enjoyable, with no negative report of burden or emotional discomfort.

## Methods

### Participants

The participants included, testimonial writers: 36 students (23 females, 13 males) of grades 10-12 (ages 16-18) from 2 rural high schools in Western New York wrote testimonials. All trained Sources of Strength peer leaders in each school (approximately 45) were invited to participate and given parent information letters and permission forms to return; and raters: 24 (10 females, 14 males) ninth grade (ages 14-15) students whose parents gave them permission to participate in this study rated on the testimonials. Two classes of ninth grade students in each school (approximately 50) were invited via to participate via a school-wide announcement and given parent information letters and permission forms to return. All participants were paid US$10 as a thank you for their time. The University of Rochester institutional review board approved this study.

### Measures

Students rated testimonials on the following dimensions using a 1-5 (‘Strongly Disagree’ to ‘Strongly Agree’) Likert scale: relatable (“My friends and I handle similar challenges”), useful/relevant (“This student’s solution/advice would work for me”), intriguing (“I am curious about more detail/background to this SPECIFIC experience”), and likable (“I would be interested in hearing more student experiences like this”).

### Design

We tested the StoryPRIME interface using a double-blind, randomized controlled design. Writers were randomly assigned to write a personal story with the aid of StoryPRIME (N=18 with 12 females) or to a control condition (N=18 with 13 females). Within each school, we assigned an equal number of students into either StoryPRIME or control condition–7 per condition for one, and 11 per condition for another school. There was no opportunity for “contamination” because both groups worked on testimonials at the same time in a quiet room on separate computers–participants simply received different on-screen instructions and interface depending upon their condition. We asked student writers to write as many testimonial as they could during a 30-minute session. Most students (N=34) completed writing 2 testimonials while 2 of them wrote just one each in the allotted time. Testimonial writers collectively produced 70 testimonials, 35 in the StoryPRIME condition and 35 in the control condition. At the end of the writing session, we asked students to complete a brief Web-based anonymous survey for their feedback on their writing experience and StoryPRIME interface.

Testimonials were presented in identical format and random order to the ninth grade student raters (N=24 with 10 females) who were blind to the writer’s condition. Each testimonial was rated by at least 3 different students. Testimonial raters rated a random selection of 12 testimonials each, 6 in each condition. Ratings were conducted anonymously on private laptops of the raters and they received payment immediately after submitting their responses. In addition to explaining to the raters that we would not know who provided which ratings, instructions emphasized that we desired and needed their honest opinions–positive and negative.

### Conditions

We met with student writers in their school’s computer lab where each student was assigned a computer. All student testimonial writers accessed a website developed for this study on which they were invited to write strength-based testimonials that would help and appeal to ninth grade students:

The Center for the Study and Prevention of Suicide works with high schools to teach teenagers emotional skills so that they stay strong when they face hard times. To do that, we need real examples from people who have used different strategies for healthy re­sponses to conflict or common challenges they face in high school.

Students then selected a challenge to write about from a list of common challenges faced by high school students, generated with the help of the student workgroup in the participatory design process (see Textbox 1).

#### StoryPRIME condition

Participants in the StoryPRIME condition were asked to:

*Think of a time in high school when you handled or learned something about* [the challenge user selected previously].

After proceeding, the participants saw:

Thank you! Now we will ask you THREE questions to help you write the details about the challenge (stress) you just selected. Please be as specific as possible. At the end, we will ask you to summarize your answers into a ~300-character story/testimonial.

**Figure 1:**
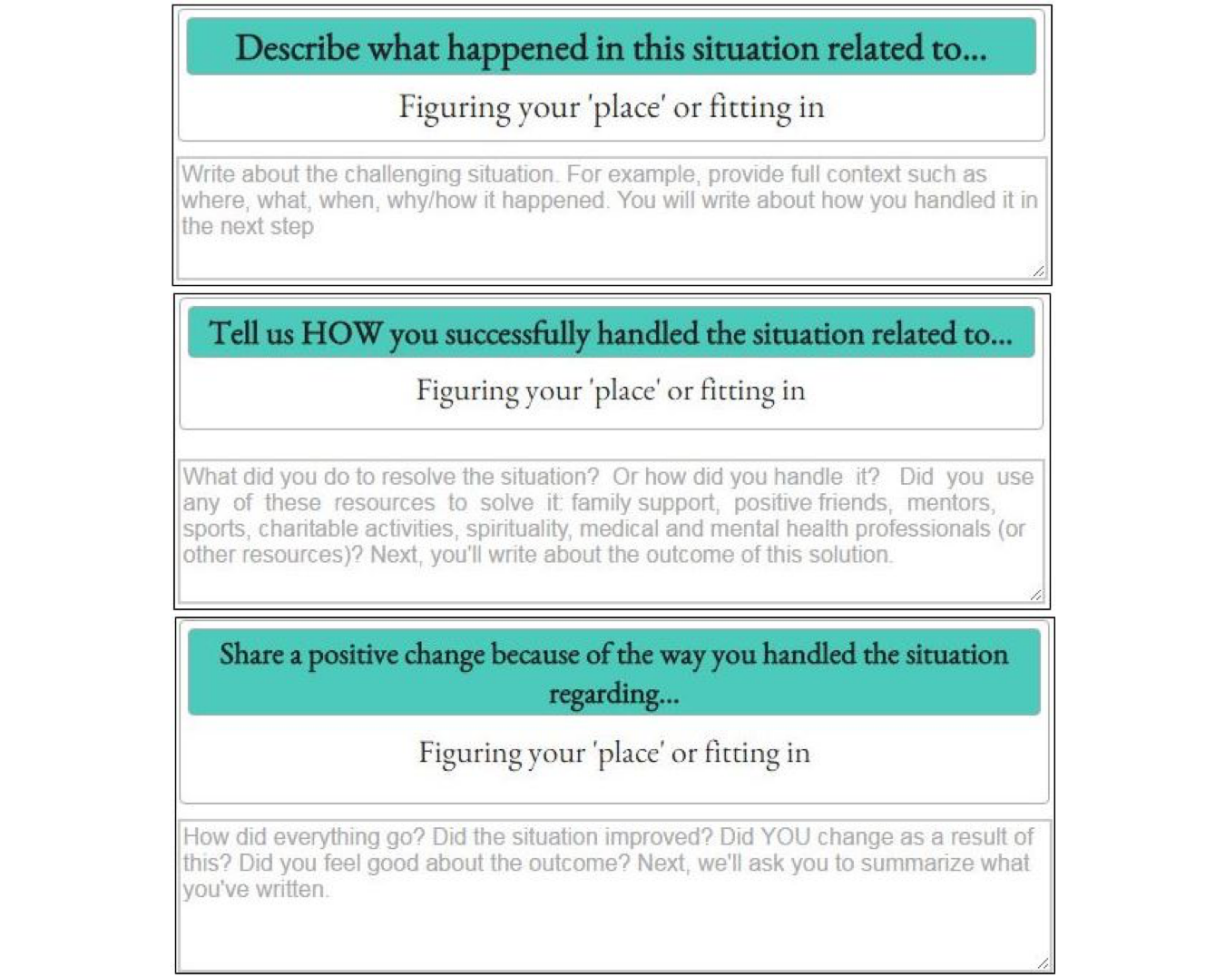
The three sequential writing prompts and corresponding clues shown to testimonial writers in the StoryPRIME condition who chose “Figuring out one’s ‘place’ or fitting in” as their challenge.

**Figure 2:**
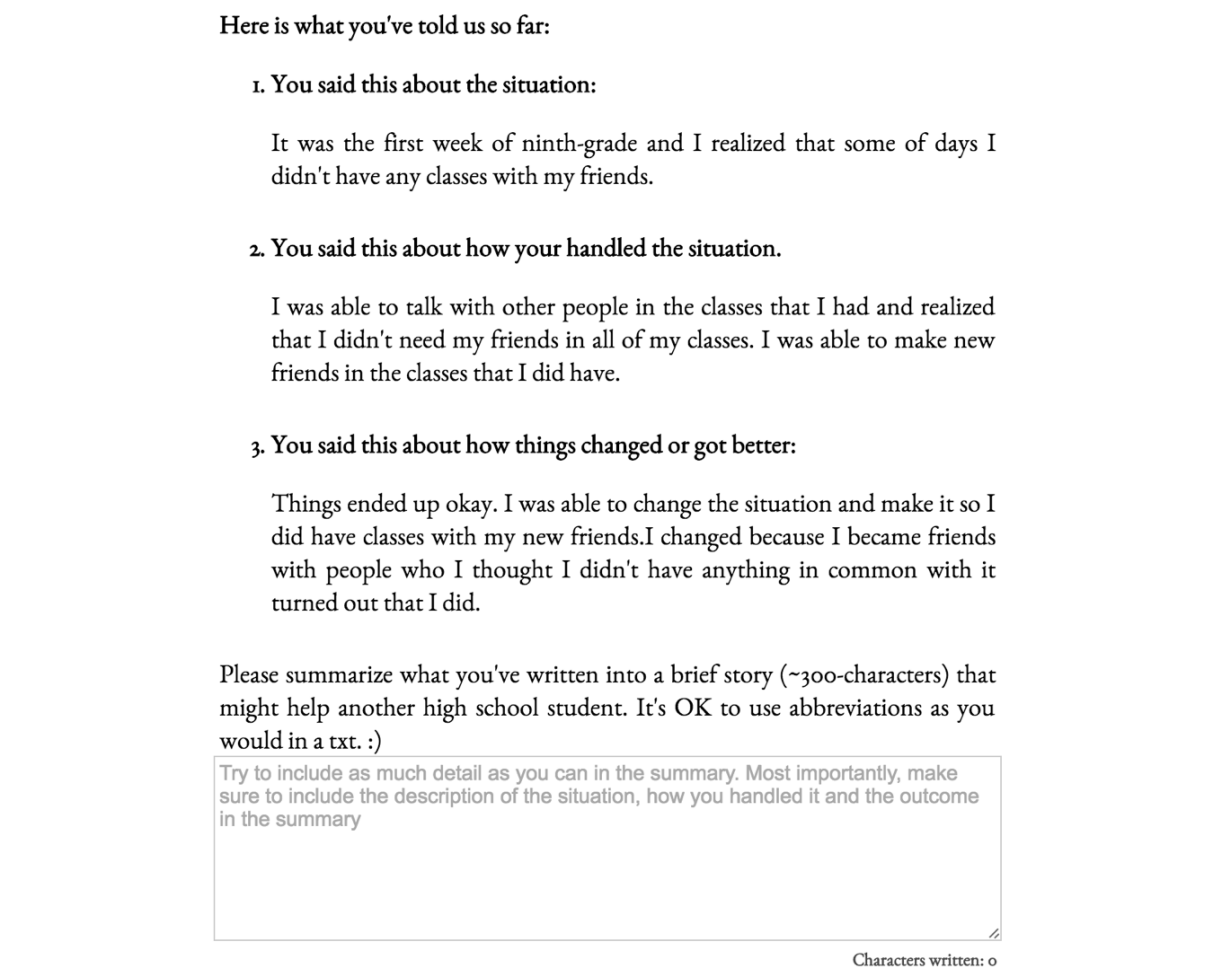
The final step of StoryPRIME in which testimonial writers are shown their responses to the earlier three writing prompts on a single page.

If the participants chose the “Ready to Help” option, they were presented with a series of 3 writing prompts and accompanying tips. Figure 1 shows these writing prompts for the challenge of “Figuring out one’s ‘place’ or fitting in.” After completing all 3 steps, participants were shown all of their previous responses on a single page and asked to generate a concise summary (∼300 characters) as shown in Figure 2. Following completion, participants were asked to repeat the procedure for another challenge.

**Common challenges (stressors) presented as options to testimonial writers, collected from the student participatory design workgroup.**

1. Learning how to balance time

2. Different class schedule from friends

3. School pressure (classes, grades, tests)

4. Not sure how to get help from teachers/adults

5. Getting cut or benched in a sport or favorite activity

6. Figuring out one’s “place” or fitting in

7. Friend or boyfriend/girlfriend drama

8. Family/parent issues

9. Feeling confused, strong feelings, ups and downs

#### Control condition

Participants in the control condition were given simple instructions: “In the space below, please share a brief story (~300 characters) about [the challenge user se­lected previously], which might help another high school student. It’s OK to use abbreviations as you would in a txt.”

### Statistical Analyses

Linear mixed-effects models were used to model the average of the 4 questions as a function of intervention (StoryPRIME vs control), controlling for rater via random effects. Similar models were used to model the Likert score for each individual question. Restricted maximum likelihood estimation via lme() in R ×64-bit version 3.1.1 was used to fit all models. We decided a priori in consultation with our biostatistician (DP) to use significance level of .10 in order to match the scientific needs of testing a novel procedure–that is, to mitigate the risk of throwing out a useful procedure based on an overly rigorous (and arbitrary) *P* level [34,35].

## Results

### Principal Findings

Ninth grade students (N=24), who were blind to the conditions, found testimonials written with the aid of StoryPRIME (N=35) more favorable than those written with the control interface (N=35). The mean of the 4 ratings–each using the 1-5 Likert scale where 5 is the maximum favorable score achievable–for testimonials written in the control condition was 3.47 (standard error (SE)=.10), and the mean for testimonials written in the StoryPRIME condition was 3.60 (SE=.10). The difference in mean ratings across conditions was .13 (SE=.08), *P*=.054. When questions were examined individually, student ratings on all 4 questions were in the same direction favoring the StoryPRIME condition over the control. As presented in Table 1, ninth grade students, who were blind to the conditions, found testimonials that had been written with the aid of StoryPRIME more relatable, useful/relevant, and likable than those written with the control interface.

**Table 1:** Summary of ratings for testimonials written in the control versus StoryPRIME conditions.

	Control mean (SE^a^)	StoryPRIME mean (SE)	Intervention effect mean (SE)	One-sided *P* value
Relatability	3.62 (.11)	3.76 (.11)	.14 (.11)	.097
Usefulness/relevance	3.48 (.10)	3.65 (.09)	.17 (.11)	.055
Intrigue	3.33 (.13)	3.42 (.13)	.09 (.09)	.165
Likability	3.44 (.13)	3.58 (.13)	.13 (.10)	.088
Mean of 4 ratings	3.47 (.10)	3.60 (.10)	.13 (.08)	.054

^a^SE: standard error.

### Student Testimonials

The challenges students wrote about centered mostly on academic pressures (grades, tests), time management, and friends. Some were about fitting in and boy/girlfriend drama. Specifically, the breakdown of peer leader testimonials was as follows: 25 testimonials related to school pressure (12 control, 13 intervention); 19 about balancing time (11 control, 8 intervention); 8 regarding different class schedules from friends (4 control, 4 intervention); 3 about feeling confused (3 control); 4 about relationship drama (2 control, 2 intervention); 6 about fitting in (2 control, 4 intervention); 1 about getting cut in sports (1 control); 3 about not knowing how to get help from teachers and adults (3 intervention); and 1 about family/parent issues (1 intervention). Some of the testimonials written in StoryPRIME can be seen in Textbox 2.

**Illustrative examples of testimonials generated by StoryPRIME.**

*Coming into high school my grades slipped and I knew I needed help but I was too scared to ask for help. After failing to keep my grades up I had to break out my shell and ask my older peers because I knew they knew how to keep their grades up and improve my time management. Their adviced helped me.* (Challenge (stressor): school pressure)

*At the start of 9^th^ grade i had difficulty handing in homework and sometimes projects. I attempted to do all my school work all at one time and it would leave me mentally exhausted. When my college success class worked on managing time, I started doing better in all my classes. Now I feel good about myself.* (Challenge: learning how to balance time)

*I didn’t have many classes with my friends one year but we made it work. I found them in the cafeteria and we still hung out outside of school. If you don’t have friends in your classes then it’s a chance to make new ones. I made a bunch of new friends and learned to talk to new people.* (Challenge: different class schedule from friends)

*After 8^th^grade, my two best friends had moved and prior to that I didn’t talk to many other people. In 9th grade I began talking to people that I normally wouldn’t have, and began involving myself in activities so I had more opportunities to meet more people. I ended up making a lot of new friends.* (Challenge: fitting in)

*My grandmother died of old Age on New Years day, at 2 am. I cried non-stop when I found out and eventually after a month of focusing on school activities and support from my my mom, I got over it and accepted it as life.* (Challenge: family issue)

The StoryPRIME interface prompts writers to describe how they applied some common coping strategies–such as family support, mentors, and positive friends as seen in the second prompt of Figure 1. Students wrote stories that mentioned relying on family support (1 in control, 2 in intervention); positive friends (5 control, 11 intervention); mentors (4 control, 14 intervention); sports (2 control, 0 intervention); and none (23 control, 8 intervention).

Most students in both conditions were happy to have shared their stories. For example, when asked:

How did you feel? Did you find anything upsetting or uncomfortable while writing the story?

One student in the control condition wrote:

It was an aha moment for me and realization.

Another student in the StoryPRIME condition wrote:

I'm happy that I was able to share my story, in order to provide advice for students who are entering high school and preparing for challenges, as well.

## Discussion

### Challenges, Achievements, and Feedback

The randomized controlled experiment produced promising results: (1) high school students assisted by StoryPRIME wrote numerous testimonials about diverse challenges such as school pressure (grades, tests), time management, friends, fitting in, and boy/girlfriend drama, (2) compared with the control condition, students assisted by StoryPRIME wrote testimonials that ninth graders found more relatable, useful/relevant, and likable, and (3) students had a positive experience writing testimonials using the StoryPRIME interface based on the overwhelmingly positive feedback they provided in the exit survey. Although the differences between the StoryPRIME testimonials and the control testimonials are small, they are meaningful–especially given the scale at which we could use StoryPRIME to reach potential student testimonial contributors.

Regarding the nonsignificant results with respect to whether messages were intriguing (“I am curious about more detail/background to this SPECIFIC experience”), we suspect that the suggested 300-character limit might not be sufficient enough to write intriguing (interesting) stories. Allowing students to write more characters may encourage them to include more details about how the event transpired, thereby making the story more intriguing. This goes in line with some suggestions from writers of both StoryPRIME and control condition such as:

Ask for more details

Add more pictures and fun questions such as how are you doing? do you feel okay today? etc.

...maybe just letting kids describe their situation more and how family was involved.

The downside of allowing more details is that the resulting stories may no longer fit the criteria of a short text message, which is what we originally set out to achieve for our text message campaign.

Feedback on StoryPRIME interface by student writers were especially encouraging; one of the writers in StoryPRIME condition wrote:

I thought this website (StoryPRIME) was great. I liked the fact that it helped me remember some times I struggled by giving me examples of things other students struggled with.

On the other hand, a writer in control condition suggested:

more room to write about something or more prompting questions for personalization.

Also, another wrote:

It's a bit hard to recall what happened over a years ago and decide which one to write about.

These comments and better ratings achieved by testimonials written using StoryPRIME suggest that the interface is helping students recall relevant topics in high school and write effective stories about them.

Despite the aforementioned positive feedback, the StoryPRIME interface is far from perfect. A few writers in StoryPRIME condition suggested potential improvements to the interface. One wrote:

You can improve this website by having us go into more depth of how we handled the situation.

Referring to the final summary step in StoryPRIME where the interface suggested students to limit their writing to 300 characters, another suggested:

(Allow) More characters to type your summary with.

Nevertheless, some of them provided positive feedback about the interface:

This website is awesome as it is :)

Honestly, I don't feel that this website needs any improvement. It is very easy to follow and helped me tell my stories.

In addition to helping students to write testimonials, StoryPRIME interface seemed to provide them with an opportunity to reflect and learn from their past experiences. One writer in StoryPRIME condition wrote:

it was easy and felt nice to describe an incident that helped me become a good student.

Another wrote:

...it was an eye opening experience. I learned that I can reflect back on my early high school career in detail.

Finally, one student wrote:

I learned that i actually learned from my mistakes as a freshmen to be more successful.

The StoryPRIME interface helped prevention researchers translate a key component of an existing in-person, evidence-based practice from a public health intervention (ie, eliciting Sources of Strength peer leaders to share their stories) onto a Web-based platform (ie, text messaging), which is a hurdle many evidence-based public health programs face as they seek to increase their reach through technology [2,36,37]. The ability to reliably generate short-form testimonials via StoryPRIME opens up new possibilities for other public health practitioners who wish to draw on peer models to deliver messages through short-form Web-based media, such as text messages and social media microblogs [38]. Ultimately, we plan to use StoryPRIME to elicit strength-based testimonials from members of the target population who are not peer leaders. By translating a school-based technique into a Web- and text messaging–based one, we hope to increase the reach of Text4Strength to an audience broader than the one reached by its parent program, Source of Strength, alone. However, it is worth nothing that in this experiment, we are testing the efficacy of the procedure in generating appealing and relevant testimonials, not the efficacy of the procedure in suicide prevention. The latter will be accomplished in a trial Text4Strength program (currently underway) to which StoryPRIME contributes.

### Limitations

Several limitations should be noted. First, this trial was conducted in 2 small, rural schools in the United States. While we have no reason to expect students from different backgrounds to respond differently from these students, the interface still needs to be tested in a variety of environments and prevention programs. Moreover, the raters in both control and intervention groups might not be representative of all ninth graders in those schools. Second, while StoryPRIME produced more relatable, useful, and likable testimonials, the effect size of the difference between the StoryPRIME and the simple control conditions was not large, so practitioners need to weigh the benefit of using StoryPRIME with the cost of a longer procedure. Finally, a nontrivial portion of the testimonials participants wrote were not interesting or relevant, and could not be used in our program. Thus, we have a procedure that generates high-quality testimonials to choose from, but have not yet arrived at the ideal state in which our interface could elicit youth testimonials ready to be automatically shared without monitoring.

### Comparison With Prior Work

While our work is the first to successfully translate an element of a school-based suicide prevention program onto a Web-based and text messaging–based platform, it builds on previous research on public health related to: (1) text messaging in public health interventions, (2) peer opinion leaders’ effectiveness in public health interventions, and (3) other public health efforts to use short form messaging. Previous work with text messaging in nonclinical settings involved smoking cessation [37], child and maternal health, and sexual health [23-25], but ours is the first effort to use texting in population-oriented suicide prevention, as we ultimately aim to do in Text4Strength. Using testimonials created with StoryPRIME in Text4Strength text messages is consistent with “Texting for Public Health Toolkit’s” strategy of crafting text messages with “engaging writing devices” [39]. While researchers have shown that peer leaders can effectively influence social norms and behavior in public health contexts, such as suicide prevention interventions [8], and in health care settings [6,7], our work is the first to translate the influence of peer leaders onto Web-based media in order to increase the reach of our intervention. This work is quite different from previous work that translated the content of a curriculum or treatment manual into text [37]. Rather, our work involved translating a complex training and coaching process that occurs in a school-based program into a Web-based interface, ultimately yielding text messages to convey public health messages. Finally, our work contributes to the growing body of research involving translating evidence-based practices into scalable Web-based formats in order to have a broader public health impact. Programs that involve community members could use a procedure like StoryPRIME to develop compelling testimonials to add to existing public health text messaging programs, such as those used increase adherence to malaria treatment [40,41].

### Conclusions

StoryPRIME serves as an example for translating complex procedures from an in-person evidenced-based program and moving them to a Web-based one in order to scale up technology use. We translated the procedure for collecting peer-to-peer testimonials, a critical component of Sources of Strength. StoryPRIME elicited high school peer leaders to generate engaging testimonials suitable for use in text messaging. The next step is to incorporate StoryPRIME testimonials into Text4Strength, a text messaging extension of Sources of Strength, to determine whether the testimonials are effective in promoting positive coping and emotional skills. Other programs that disseminate peer testimonials in public health messaging can draw on this work to improve the efficiency and effectiveness of collecting brief testimonials for use in short-form, Web-based media, such as text messages and social media microblogs.

## Abbreviations

HCI: human-computer interaction
HIV: human immunodeficiency virus
MTurk: Mechanical Turk
StoryPRIME: stories of personal resilience in managing emotion

## References

[1] Norman CD, Haresign H, Mehling C, Bloomberg H (2016). Exploring the feasibility and potential of virtual panels for soliciting feedback on nutrition education materials: a proof-of-concept study. JMIR Public Health Surveill.

[2] Rempel GR, Ballantyne RT, Magill-Evans J, Nicholas DB, Mackie AS (2014). Texting teens in transition: the use of text messages in clinical intervention research. JMIR Mhealth Uhealth.

[3] Sze YY, Daniel TO, Kilanowski CK, Collins RL, Epstein LH (2015). Web-based and mobile delivery of an episodic future thinking intervention for overweight and obese families: a feasibility study. JMIR Mhealth Uhealth.

[4] Sikkema KJ, Kelly JA, Winett RA, Solomon LJ, Cargill VA, Roffman RA, McAuliffe TL, Heckman TG, Anderson EA, Wagstaff DA, Norman AD, Perry MJ, Crumble DA, Mercer MB (2000). Outcomes of a randomized community-level HIV prevention intervention for women living in 18 low-income housing developments. Am J Public Health.

[5] Valente TW, Chou CP, Pentz MA (2007). Community coalitions as a system: effects of network change on adoption of evidence-based substance abuse prevention. Am J Public Health.

[6] Schneider JA, Cornwell B, Ostrow D, Michaels S, Schumm P, Laumann EO, Friedman S (2013). Network mixing and network influences most linked to HIV infection and risk behavior in the HIV epidemic among black men who have sex with men. Am J Public Health.

[7] Schneider JA, Zhou AN, Laumann EO (2015). A new HIV prevention network approach: sociometric peer change agent selection. Soc Sci Med.

[8] Wyman PA, Brown CH, LoMurray M, Schmeelk-Cone K, Petrova M, Yu Q, Walsh E, Tu X, Wang W (2010). An outcome evaluation of the Sources of Strength suicide prevention program delivered by adolescent peer leaders in high schools. Am J Public Health.

[9] Bock BC, Rosen RK, Barnett NP, Thind H, Walaska K, Foster R, Deutsch C, Traficante R (2015). Translating behavioral interventions onto mHealth platforms: developing text message interventions for smoking and alcohol. JMIR Mhealth Uhealth.

[10] Christakis NA, Fowler JH (2008). The collective dynamics of smoking in a large social network. N Engl J Med.

[11] Pisani AR, Wyman PA, Petrova M, Schmeelk-Cone K, Goldston DB, Xia Y, Gould MS (2013). Emotion regulation difficulties, youth-adult relationships, and suicide attempts among high school students in underserved communities. J Youth Adolesc.

[12] Petrova M, Wyman PA, Schmeelk-Cone K, Pisani AR (2015). Positive-themed suicide prevention messages delivered by adolescent peer leaders: proximal impact on classmates' coping attitudes and perceptions of adult support. Suicide Life Threat Behav.

[13] LoMurray M (2005). Sources of Strength facilitators guide: suicide prevention peer gatekeeper training.

[14] Forbes E, Dahl R (2005). Neural systems of positive affect: relevance to understanding child and adolescent depression?. Dev Psychopathol.

[15] Gratz KL, Roemer L (2004). Multidimensional assessment of emotion regulation and dysregulation: development, factor structure, and initial validation of the difficulties in emotion regulation scale. J Psychopathol Behav Assess.

[16] Jacobson CM, Marrocco F, Kleinman M, Gould MS (2011). Restrictive emotionality, depressive symptoms, and suicidal thoughts and behaviors among high school students. J Youth Adolesc.

[17] Neumann A, van Lier PAC, Gratz KL, Koot HM (2010). Multidimensional assessment of emotion regulation difficulties in adolescents using the Difficulties in Emotion Regulation Scale. Assessment.

[18] Lenhart A (2012). Teens, smartphones &amp; texting. Pew Internet & American Life Project.

[19] Aguilera A, Muñoz RF (2011). Text messaging as an adjunct to CBT in low-income populations: a usability and feasibility pilot study. Prof Psychol Res Pr.

[20] Cole-Lewis H, Kershaw T (2010). Text messaging as a tool for behavior change in disease prevention and management. Epidemiol Rev.

[21] Arora S, Peters AL, Agy C, Menchine M (2012). A mobile health intervention for inner city patients with poorly controlled diabetes: proof-of-concept of the TExT-MED program. Diabetes Technol Ther.

[22] Mutsuddi A, Connelly K (2012). Text messages for encouraging physical activity Are they effective after the novelty effect wears off?. http://ieeexplore.ieee.org/document/6240360/.

[23] Daniels A, Taylor S, Post S, Pilsner A, Hunt Y, Augustson E (2012). Tech to treat: the smokefree teen approach to cessation. https://cdc.confex.com/cdc/nphic12/webprogram/Paper31682.html.

[24] Parker RM, Dmitrieva E, Frolov S, Gazmararian JA (2012). Text4baby in the United States and Russia: an opportunity for understanding how mHealth affects maternal and child health. J Health Commun.

[25] Levine D, McCright J, Dobkin L, Woodruff AJ, Klausner JD (2008). SEXINFO: a sexual health text messaging service for San Francisco youth. Am J Public Health.

[26] (2013). Results from the 2012 National Survey on Drug Use and Health: Summary of National Findings. US Department of Health and Human Services.

[27] Casey BJ, Jones RM, Levita L, Libby V, Pattwell SS, Ruberry EJ, Soliman F, Somerville LH (2010). The storm and stress of adolescence: insights from human imaging and mouse genetics. Dev Psychobiol.

[28] Hare TA, Tottenham N, Galvan A, Voss HU, Glover GH, Casey BJ (2008). Biological substrates of emotional reactivity and regulation in adolescence during an emotional go-nogo task. Biol Psychiatry.

[29] Somerville LH, Casey BJ (2010). Developmental neurobiology of cognitive control and motivational systems. Curr Opin Neurobiol.

[30] Di MA, Uddin LQ, Shehzad Z, Gee DG, Reiss PT, Margulies DS, Castellanos FX, Milham MP, Kelly A M Clare (2009). Development of anterior cingulate functional connectivity from late childhood to early adulthood. Cereb Cortex.

[31] Wilson BJ (2007). Designing media messages about health and nutrition: what strategies are most effective?. J Nutr Educ Behav.

[32] Lim MSC, Wright C, Hellard ME (2014). The medium and the message: fitting sound health promotion methodology into 160 characters. JMIR Mhealth Uhealth.

[33] Muller M, Sears A, Jacko J (2009). Participatory design: the third space in HCI. Human-Computer Interaction: Development Process.

[34] Lancaster GA, Dodd S, Williamson PR (2004). Design and analysis of pilot studies: recommendations for good practice. J Eval Clin Pract.

[35] Akaliyski P How can I justify the use of statistical significance at the 10%?.

[36] Watts S, Mackenzie A, Thomas C, Griskaitis A, Mewton L, Williams A, Andrews G (2013). CBT for depression: a pilot RCT comparing mobile phone vs. computer. BMC Psychiatry.

[37] Bock B, Heron K, Jennings E, Morrow K, Cobb V, Magee J, Fava J, Deutsch C, Foster R (2013). A text message delivered smoking cessation intervention: the initial trial of TXT-2-Quit: randomized controlled trial. JMIR Mhealth Uhealth.

[38] Young SD, Cumberland WG, Lee S, Jaganath D, Szekeres G, Coates T (2013). Social networking technologies as an emerging tool for HIV prevention: a cluster randomized trial. Ann Intern Med.

[39] Karasz H, Bosslet L (2015). Northwest Center for Public Health Practice and Seattle and King County Public Health.

[40] Jones COH, Wasunna B, Sudoi R, Githinji S, Snow RW, Zurovac D (2012). “Even if you know everything you can forget”: health worker perceptions of mobile phone text-messaging to improve malaria case-management in Kenya. PLoS One.

[41] Zurovac D, Sudoi RK, Akhwale WS, Ndiritu M, Hamer DH, Rowe AK, Snow RW (2011). The effect of mobile phone text-message reminders on Kenyan health workers' adherence to malaria treatment guidelines: a cluster randomised trial. Lancet.

